# Improved biogas production and biodegradation of oilseed rape straw by using kitchen waste and duck droppings as co-substrates in two-phase anaerobic digestion

**DOI:** 10.1371/journal.pone.0182361

**Published:** 2017-08-02

**Authors:** Chuqiao Wang, Feng Hong, Yong Lu, Xianning Li, Hengming Liu

**Affiliations:** 1 School of Energy and Environment, Southeast University, Nanjing, China; 2 Ocean Science and Environment College, Dalian ocean University, Dalian, China; Zhejiang University, CHINA

## Abstract

Oilseed rape straw (ORS) is a kind of biorefractory waste widely existing in the rural area of China, which is highly suitable to mix with kitchen waste (KW) and duck droppings (DD) in two-phase anaerobic digestion (AD). This research introduced the importance of KW and DD addition to improve the biogas production and biodegradation of ORS. A set of comparative experiments were conducted on two-phase mono- and co-digestion with organic load of 60 g VS/L. The total methane yield (TMY) and the biodegradation of ORS of co-digestions were obviously improving, and the synergistic effect found in the two-phase co-digestions. The optimum mixing ratio of ORS, KW and DD was 50:40:10, and the corresponding TMY and VS degradation rate of ORS were 374.5 mL/g VS and 49.7%, respectively. Addition of KW and DD maintained the pH within the optimal range for the hydrolyzing-acidification, improved the phase separation and buffering capacity of AD system.

## 1. Introduction

The oilseed rape is one of the most important oil-crops in China, and the annual oilseed rape straw (ORS) production reaches 20 million tons. The Yangtze River Basin is the main growing region of rapeseed with the yield accounting for more than 90% of the total in China. These areas produce abundant ORS every year, most of which were burned after harvest, which not only waste resource but also cause pollution to the air and the surroundings.

Due to the current fossil energy resources crisis [1], anaerobic digestion (AD), which converted organic matter in the ORS into biogas and organic fertilizer, has been proved to be an economic and clean method. However, the ORS has high lignin content and carbon/nitrogen (C/N) ratio, which limits the anaerobic biodegradation process. Some researchers [2, 3] found that lignin content in organic substrates could lead to low biogas yield and low degradation rate. Therefore, there were only a few researches using ORS as feedstock for AD [4, 5]. However, the batch feeding mode is usually used for the straw fermentation on account of the hard-degraded lignocellulose contains in straw. The straw has adsorption and interception effects to the microorganism that can cause sludge loss when reloading the digester. To ensure the subsequent digestion well, the new inoculated sludge should be fed, which caused extension of fermentation time and increase of the operating costs. The large amounts of inoculum used in single phase AD can be greatly reduced in two-phase AD.

Recently, many studies have shown that co-digestion is an attractive approach for improving biogas production and biodegradation efficiency [6, 7]. Kitchen waste (KW), and duck droppings (DD) are both readily available typical organic solid wastes with a large amount of production in rural areas in China [8]. KW contains high content of readily biodegradable compositions and is easily converted to biogas, but it has a low buffering capacity. However, the KW containing Na^+^ and other cationic elements could show inhibitory effects on microorganisms when the concentration increased to a high level [9], so it is improper to feed high proportion of KW in anaerobic digester. Many studies showed that digestion of materials with high concentration of nitrogen has low C/N, such as animal manure, easily leading to ammonium nitrogen accumulation, digestion inhibition, and low biogas production rate [10, 11]. Based on earlier studies, it suggests that compared with mono-digestion, the use of kitchen waste [7] and livestock manure [12] as co-substrates with crop straw can improve biogas production from AD.

Most of these studies were conducted with regards to co-digestions of crop straws using single phase anaerobic digester [7, 12]. The single-phase co-digestions need more animal manure to maintain suitable pH for biogas production, increasing the risk of ammonium nitrogen inhibition accumulation [13]. More recently, two-phase AD gained more and more attention due to many advantages, such as high buffering capacity and easily reloading substrates of the hydrolytic-acidification reactor (HAR) in batch mode. Most of these studies were focused on co-digestions of crop straws with single types of substrates and conducted in single phase AD to improve the biogas production [14, 15]. However, according to the literature survey, no previous study has investigated the two-phase anaerobic co-digestion of ORS, KW and DD. The synergistic effects and optimal mixing combinations between ORS, KW and DD are not clear.

Hydrolysis step is the limiting step of ORS in AD [16]. Some studies [17] used reduction in VS to evaluate the efficiency of anaerobic digestion, and draw conclusions that higher rate of VS destruction was obtained in co-digestion than in mono-digestion. It is apparent that, the readily biodegradable materials in co-substrates could improve the VS destruction rate of the overall digestion systems [8]. Previous studies pay more attention to the biodegradation of the overall substrate in co-digestion, but do not pay much attention to the biodegradation enhancement of straws by adding co-substrates in two-phase AD [17, 18]. Therefore, the combined effect of ORS biodegradation by using KW, DD as co-substrates in two-phase AD should be further studied.

The viability of co-digestion ORS, KW and DD by two-phase anaerobic reactor in batch mode were presented in this study. The objectives of this study were (1) to evaluate the biogas production of co-digestions and mono-digestions in two-phase mesophilic anaerobic digester and demonstrate the synergistic effects of co-substrates and ORS, (2) to optimize the best ratio in the different ORS/co-substrates mixtures on account of the biogas production, ORS degradation and changes in hydrolysis-acidogenesis liquid, and (3) to investigate whether using DD and KW as co-substrates with ORS in two-phase anaerobic digester may improve the ORS biodegradation.

## 2. Materials and methods

### 2.1. Raw substrates and inoculum

The field study was permitted by administration committee of the Zheng Pu port development zone. ORS, KW, DD and inoculum used in this study were sourced from Muchang Village, MuqiaoTownship, Maanshan, Anhui, China. ORS were collected from the oilseed rape field after harvest which was air-dried, chopped, and screened to 18 mesh (1–1.18 mm). KW was collected from kitchens of a modernized rural communities (equipped with a refuse classification system), which was mixed and smashed by food machine (Foshan, China) and frozen at -20°C. DD was collected from a duck farm near the village which was preserved at -20°C after feather removal. Inoculum was collected from a household anaerobic digester treating swine manure at local village. Sludge was acclimated, which used mixed leachate of ORS, KW and DD (the proportion was 50:25:25) in laboratory anaerobic digester at 35°C for 5 months before utilization. The main characteristics and compositions of these substrates and inoculum are summarized in Table 1.

**Table 1:** Characteristics of raw substrates and inoculum.

Characteristic	Oilseedrape straw	Kitchen waste	Duck droppings	Inoculum
pH	ND	3.85±0.02	8.54±0.11	7.97±0.13
Total solids, TS(%WW^a^)	87.97±0.56	29.26±0.51	37.00±0.78	4.68±0.15
Volatile solids, VS(%WW^a^)	78.34±0.53	26.78±0.52	25.24±0.41	2.51±0.08
VS/TS ratio (%)	89.05±0.54	91.52±0.78	68.25±1.83	53.68±0.63
Carbon, C(%TS)	40.87±0.54	51.16±0.31	29.53±0.21	ND
Nitrogen, N(%TS)	0.81±0.37	2.08±0.14	2.81±0.14	ND
Hydrogen, H(%TS)	5.77±0.44	8.52±0.21	4.84±0.19	ND
Oxygen, O(%TS)	41.18±0.49	29.27±0.51	30.71±0.31	ND
C/N ratio	50.4±7.2	24.6±2.1	10.5±1.7	ND
Cellulose(%TS)	42.62±1.83	16.60±2.36	22.44±2.82	ND
Hemicelluloses(%TS)	21.21±1.21	6.9±1.72	11.84±1.39	ND
Lignin(%TS)	23.15±1.66	3.0±1.21	0.97±0.58	ND
Ash(%WW^a^)	9.63±0.51	2.48±0.45	11.75±0.88	2.17±0.20
TVFA^b^(g L^-1^)	ND	4.78±0.08	0.43±0.02	ND
TA^c^(gCaCO_3_ L^-1^)	ND	0	9.22±0.21	7.44±0.12
NH_4_^+^-N^d^(g L^-1^)	ND	0.51±0.03	3.14±0.08	ND

Data are the averages of three measurements, and numbers after plus-minus signs are the standard deviations.

^a^ WW: wet weight.

^b^ TVFA: total volatile fatty acids.

^c^ TA: total alkalinity.

^d^ NH_4_^+^-N: ammonia nitrogen.

### 2.2 Operation of two-phase anaerobic digestion

**Fig 1:**
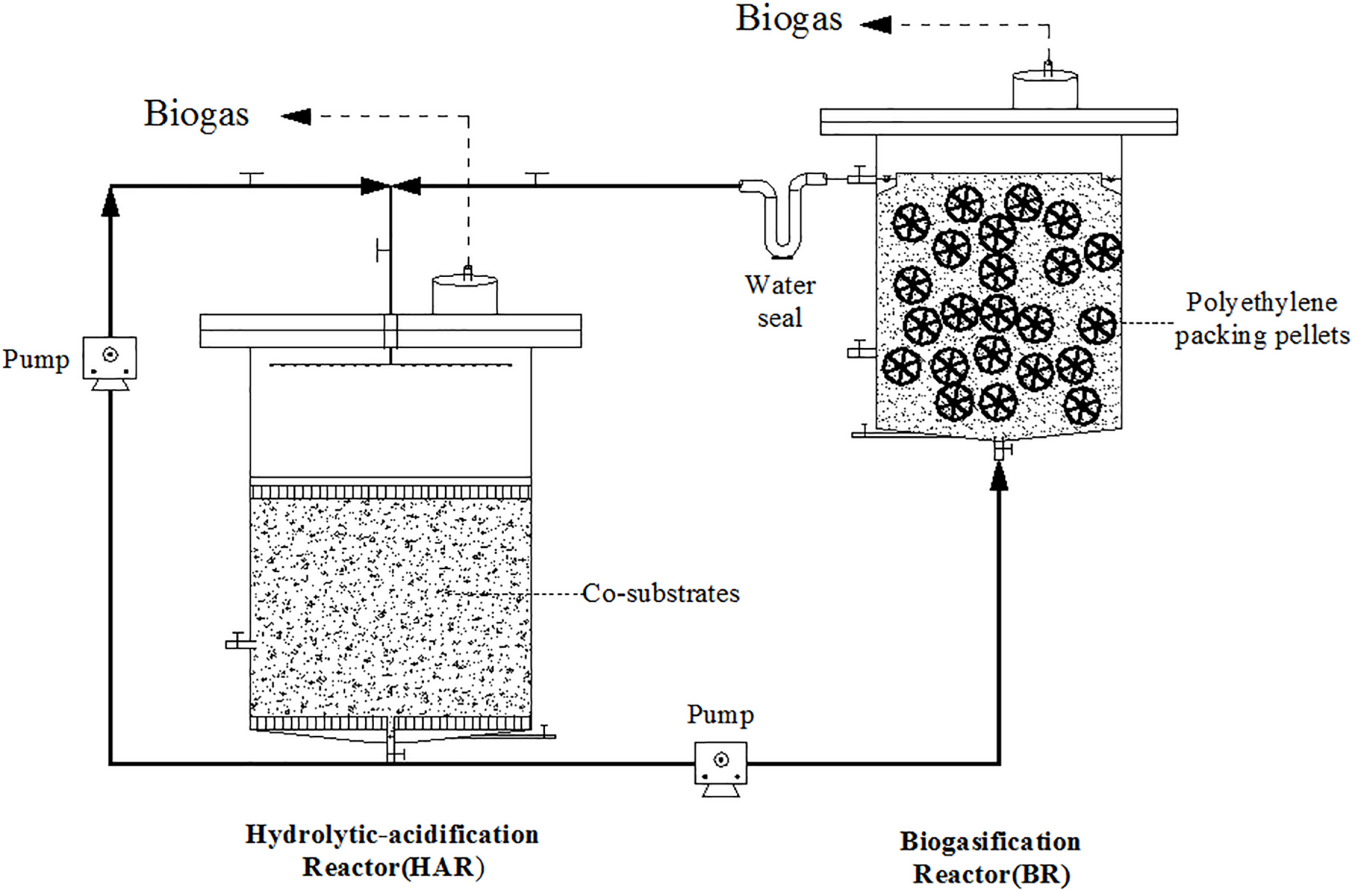
Schematic of a batch two-phase digester for the treatment of co-substrates.

The two phase anaerobic digestion system (Fig 1) consisted of a 15 L hydrolytic-acidification reactor (HAR) and a 5.6 L biogasification reactor (BR), which working volume were 8 L and 4 L, respectively. Each reactor was made of transparent acrylic. The solid-bed reactor is designed for the hydrolysis and acidification, and connected to the bottom of upflow anaerobic filter reactor which is designed for the methane fermentation. The polyethylene packing pellets were suspended in the BR of 2.5 L to intercept microorganism and provide sufficient surface area for the attachment of more microorganism. Six HARs were submerged in a 35±1°C water bath, and internal heat exchangers were used to keep six BRs at 35±1°C. The 35±1°C water in heat exchangers was provided by water bath and circulated by magnetic pump.

The inoculum was taken from a two-phase anaerobic reactor (60 L) running steadily in laboratory, and then quickly concentrated and transferred into BRs (5.6 L) of two-phase anaerobic digesters. The weights of sludge added into BRs were 160 g in all based on VS. The leachate of ORS, KW and DD were preliminarily used to acclimatize anaerobic bacteria before the digestion for 15 days. Except for mono-digestion group, the proportion of ORS in each HAR was kept 50% (30 g VS/L, VS loading) while the KW and DD were added at variable proportion. The proportions (based on VS loading) of KW or DD in two-phase co-digestion (G1 and G2) were designed as 50% and 50%, respectively. The ORS: KW: DD ratios of groups G3, G4, G5 with three kinds of substrates were designed as 50:40:10, 50:25:25 and 50:10:40, respectively. The ORS percentage fixed in 50% is because there are abundant ORS produce in rural area of China, but the amounts of KW and DD are relatively few. In order to promote hydrolysis-acidification rate, the fermentation slurry in HAR (70 L) of laboratory two-phase anaerobic reactor was diluted (TVFA 17.1±1.2 mg/L), and then added into HAR to the designed volume. All reactors were purged with N_2_ gas for 3 min to expel the oxygen before being sealed.

The acrylic perforated plate (4 mm holes), which is covered by nylon mesh (0.8 mm), and put at the bottom of the solid-bed reactor to separate substrates from leachate. The substrates mixtures were processed in batches, and the digestion was operated in a batch mode with solid retention time of 35 days. The previous laboratory experiments showed the increasing trends of TVFAs concentration in HARs became gentle after the 6th day. After mono- or co-substrates were fed in HARs for the first 6 days, the HARs were disconnected to the corresponding BRs, and the liquid inside was self-circulated using peristaltic pump at 11.1 mL/min for 30 min intermittently, 24 times per day. From the 7th day on, leachates of the HARs were pumped into corresponding BRs at 5.6 mL/min for 20 min intermittently, 12 times per day. Reflux liquid from BR to HAR was flowed through a U-shape water seal pipe by gravity flow.

### 2.3 Theoretical and weighted specific methane yield

The theoretical methane potential (TMP) of organic substrates can be estimated by the Buswell formula based on elemental composition, as shown in Eqs 1 and 2.

$$C_{n}H_{a}O_{b}N_{c}+(n-\frac{a}{4}-\frac{b}{2}+\frac{3c}{4})H_{2}O\to (\frac{n}{2}+\frac{a}{8}-\frac{b}{4}-\frac{3c}{8}){CH}_{4}+(\frac{n}{2}-\frac{a}{8}+\frac{b}{4}+\frac{3c}{8}){CO}_{2}+{cNH}_{3}$$ (1)

$$TMP\left(\frac{mL{CH}_{4}}{gVS}\right)=\frac{22.4\times 1000\times (\frac{n}{2}+\frac{a}{8}-\frac{b}{4}-\frac{3c}{8})}{12n+a+16b+14c}$$ (2)

The weighted average methane content (WAMC) was calculated based on Eq 3. The weighted specific methane yield (WSMY) was calculated as the weighted average of the individual substrates’ total methane yield (TMY).

$$\text{WAMC}(\%)=\frac{\sum_{\text{i}=1}^{\text{n}}{\text{BP}}_{\text{i}}\times {\text{MP}}_{\text{i}}}{\sum_{\text{i}=1}^{\text{n}}{\text{BP}}_{\text{i}}}$$ (3)

where: BP_i_ = biogas production on day i,

MP_i_ = methane content on day i,
n = number of digestions.

### 2.4 Biodegradation of ORS

At the end of the anaerobic digestion, the HARs were drained out, and the VS and content of CHL (cellulose, hemicellulose and lignin) of fermented ORS were determined. The degradation rate of ORS and removal rate of the CHL of fermented ORS were calculated as Eq 4 and Eq 5, respectively.

$$\mathrm{Degradation}\;\mathrm{rate}\;\mathrm{of}\;\mathrm{ORS(\%)}=\frac{\mathrm{VS}\;\mathrm{of}\;\mathrm{ORS}\;\mathrm{before}\;\mathrm{AD}-\mathrm{VS}\;\mathrm{surplus}\;\mathrm{after}\;\mathrm{AD}}{\mathrm{VS}\;\mathrm{of}\;\mathrm{ORS}\;\mathrm{before}\;\mathrm{AD}}\times 100$$ (4)

$$\mathrm{Removal}\;\mathrm{rate}\;\mathrm{of}\;\mathrm{CHL}(\%)=\frac{\mathrm{CHL}\;\mathrm{in}\;\mathrm{ORS}\;\mathrm{before}\;\mathrm{AD}-\mathrm{CHL}\;\mathrm{in}\;\mathrm{ORS}\;\mathrm{after}\;\mathrm{AD}}{\mathrm{CHL}\;\mathrm{in}\;\mathrm{ORS}\;\mathrm{before}\;\mathrm{AD}}\times 100$$ (5)

### 2.5 Analytical methods

Moisture content (MC), Total solids (TS) and volatile solids (VS) of ORS, KW and DD, before and after digestion, were determined according to Standard Methods [19]. Elemental composition of the ORS, KW and DD were measured by elemental analyzer (CHN-O-Rapid, Heraeus, Germany). Hemicellulose, cellulose, and lignin content were determined according to the method described by Van Soest [20]. Each solid or semisolid sample was analyzed in triplicate.

Liquid samples were taken from each hydrolytic-acidification digester daily during the first 6 days of the batch digestion, and from 7th day, each HAR and BR was sampled every 2 or 3 days. The liquid samples were primarily centrifuged at 10,000 rpm for 10 min and filtered through 0.45 μm membrane filter to remove particulate, soluble chemical oxygen demand (sCOD) and total ammonia nitrogen (TAN) were determined according to Standard Method [19]. Total alkalinity (TA) was measured by pH titration to 4.3[19]. pH was measured by pH meter (PB-10, Sartorius, Germany).Each liquid samples were analyzed in triplicate, all the residue liquid samples were returned to the corresponding reactors after analysis. The volatile fatty acids (VFAs) in HARs and BRs were measured acetic, propionate, iso-butyric, butyric, valeric and iso-valeric acid by GC (GC-2001, Shimadzu, Japan) equipped with hydrogen flame ionization detector (FID) using Nitrogen as a carrier gas in a quartz capillary column (30 m×0.32 mm×D×0.25 μm; Stabilwax-DA, RESTEK, USA). The GC was operated when the injection port and detector temperatures were 220°C and 230°C, respectively. The oven temperature-rising program was as follows: the temperature was maintained at 90°C for 1 min, increased to 130°C at rate 12°C/min and kept 2 min, and increased to190°C at rate 10°C/min and maintained 2 min.

Biogas samples were collected with gas collecting bag (E-Switch, Shenyuan, China) daily. Silicone tube was used to connect gas collected bag to wet gas-flow meter for calculating biogas volume. Methane content of biogas samples was measured by fast biogas analyzer equipped with infrared detector (GT901-CH_4_, Shenzhen, China).

### 2.6 Data analysis and statistics

Biogas yield changed constantly during the whole batch digestion process. The methane content and the biogas yields change considerably at different digestion time, which is more accurate to represent methane content in the whole batch digestion than the average only. Therefore, the weighted average methane content (WAMC) was used to depict the methane changes. The WAMC was calculated according to the introduction by El-Mashad et al (Eq 3) [21].

The measurements were conducted in triplicates and expressed as means ±standard deviation (SD). The significant differences between 8 groups were determined using one-way analysis of variance (ANOVA), the significant level (p value) was set at 0.05 and 0.01. The Duncan's multiple range test was used to analyze statistically significant differences between pairs of means with the threshold values of 0.05 and 0.01 significance level. The OriginPro 8.5 (OriginLab, USA) and SPSS19.0 (IBM, USA) were used for graphing and data processing.

## 3. Results and discussion

### 3.1 Substrate characterization

The characteristics of raw substrate, oilseed rape straw (ORS), kitchen waste (KW) and duck droppings (DD) are shown in Table 1. The VS/TS ratio is an indicator to evaluate the organic content in substrate. Based on the total weight basis, VS/TS ratio of ORS, KW and DD were 89.05, 91.52, and 68.25%, respectively. Generally speaking, substrates with higher VS/TS ratio contain more organics and more suitable for biogas production [22, 23]. However, previous study showed oilseed rape straw with high lignin content was inefficient to be converted into biogas by AD [5].The ORS used in this study contained higher lignin content (23.15%±1.66) than KW and DD, meaning that ORS was hard to be biodegraded in the AD, which was similar to those reported[4]. The C/N ratio of substrate is an important factor on biogas production in AD. The C/N ratio of ORS (50.4±7.2) was much higher than that of KW (24.6±2.1) and DD (10.5±1.7). Previous study showed the C/N ratio in the range of 15–30 was optimal for the biogas production [8]. This funding implied that the addition of KW and DD as co-substrates with ORS reduced the C/N ratio to the proper range in two-phase AD.

### 3.2 Biogas and methane production from mono-digestion

The results of three mono-substrates in the two-phase anaerobic digestion are shown in Table 2. For ORS and DD, the total biogas yields (TBYs) were 244.1 and 281.8 mL/g VS, respectively; the total methane yields (TMYs) were 132.3 and 159.2 mL/g VS, respectively. These results are similar to those from single-phase anaerobic digestion of ORS [4] and from single-phase anaerobic digestion of poultry slurry [24]. This indicated that two-phase anaerobic digestion cannot increase biogas production for individual ORS and DD.

**Table 2:** Experimental design and results of two phase co-digestion under different mono- and co-substrates.

Group unit	G1			G2	G3	G4	G5	G6	G7	G8
Operation conditions										
Digestion type	Co-			Co-	Co-	Co-	Co-	Mono-	Mono-	Mono-
ORS:KW:DD proportion	50:50:0			50:0:50	50:40:10	50:25:25	50:10:40	100:0:0	0:100:0	0:0:100
ORS g VS/L	30			30	30	30	30	60	0	0
KW g VS/L	30			0	24	15	6	0	60	0
DD g VS/L	0			30	6	15	24	0	0	60
Initial C/N	37.5			30.5	36.1	33.9	31.9	50.4	24.6	10.5
Digestion performances										
TBY^a^ mL/g VS	538.1			307.0	601.8	448.9	380.4	244.1	540.6	281.8
Biogas from HAR mL/g VS	309.1			202.2	358.2	276.3	249.6	162.5	325.5	183.6
Biogas from BR mL/g VS	229.0			104.8	243.6	172.6	130.9	81.6	240.9	98.2
WAMC^b^%	57.3			54.5	61.6	61.5	59.3	52.7	60.3	54.3
TMP^c^ mL/g VS	550.6			431.4	526.7	491.1	455.2	440.7	660.5	422.1
TMY^d^mL/g VS	317.8			171.0	374.5	275.7	231.4	132.3	335.6	159.2
TMY/TMP	0.58			0.40	0.71	0.56	0.51	0.30	0.51	0.38
Methane from HAR mL/g VS	183.6			109.8	222.8	167.8	144.8	89.9	173.5	107.3
Methane from BR mL/g VS	134.2			61.1	151.6	107.9	86.5	42.3	162.1	51.9
Methane from HAR/BR ratio	1.37			1.80	1.47	1.55	1.67	2.12	1.07	2.06
WSMY^e^mL/g VS	233.9			145.8	216.3	189.9	163.4	-	-	-
Differential^f^ mL/g VS	83.8			25.3	158.2	85.9	68.0	-	-	-

Co- and Mono- represent the co-digestion and mono-digestion, respectively.

^a^ TBY: total biogas yield.

^b^ WAMC: weighted average methane content.

^c^ TMP: theoretical methane potential.

^d^ TMY: total methane yield.

^e^ WSMY: weighted specific methane yield.

^f^ Differential: calculated by TMY minus WSMY.

The pH in all trials was not adjusted except G7 (mono-KW), in which biogasification was inhibited by low pH. Therefore, BR of mono-KW group was using NaHCO_3_ to adjust pH to 7.0 at the 16th day, and after that the pH was adjusted every 2 days. Higher total biogas and methane yield were obtained in G7, 540.6 and 335.6 mL/g VS, compared to G6 and G8, which mainly contained higher readily- biodegradable organic content in KW. On the contrary, ORS, which contains high proportion of organics showed lowest TBY and TMY. This could be attributed to the high lignin content in ORS [8, 25].

For the individual two-phase AD of ORS, KW or DD, the weighted average methane content (WAMC) were 52.7, 60.3 and 54.3%, respectively; the theoretical methane potential (TMP) were 440.7, 660.5 and 422.1 mL/g VS, respectively; the ratio of total methane yield (TMY) to TMP were 0.30, 0.51 and 0.38, respectively, which indicated that ORS was more refractory compared with KW and DD. The TMY of ORS were lower than other crop straws of previous study, this is mainly because the higher lignin content in ORS compare with others [13]. However, the TMY/TMP ratio of G7 was somewhat lower than previous results for individual KW during two-phase fermentation [26, 27]. This might be due to the late pH adjustment (compare to pH adjustment throughout digestion) and shorter digestion time in this study.

### 3.3 Biogas and methane production from co-digestion

Table 2 shows the BYs (biogas yields), MYs (methane yields),WAMC (weighted average methane content)and TMP (theoretical methane potential)of corresponding HAR, BR and overall digestion system at different mixing ratio of ORS mixed with KW and DD. For different co-digestions, the TBYs (Total biogas yields) of G1 to G5 were calculated as 538.1, 307.0, 601.8, 448.9, 380.4 mL/g VS, respectively; the TMYs (Total methane yields) of G1 to G5 were calculated as 317.8, 173.4, 374.5, 275.7, 230.7 mL/g VS, respectively. Compared with the two-phase fermentation of individual ORS, the TBYs and TMYs of co-digestions were enhanced from 25.8 to 146.5% and from 31.1 to 183.1%, respectively. Correspondingly, the highest TBY and TMY during the 36 days were both obtained in the G3. Linear increase of both TBYs and TMYs was evident between co-digestion and mono-digestion of ORS (*p*<0.05). This implied that adding KW and DD as co-substrates for ORS would effectively enhance the methane and biogas production. It is reported similarly that the addition of food waste or livestock manures as co-substrate in anaerobic digester could improve both the biogas production rates and the cumulative biogas production yield [28, 29].

**Fig 2:**
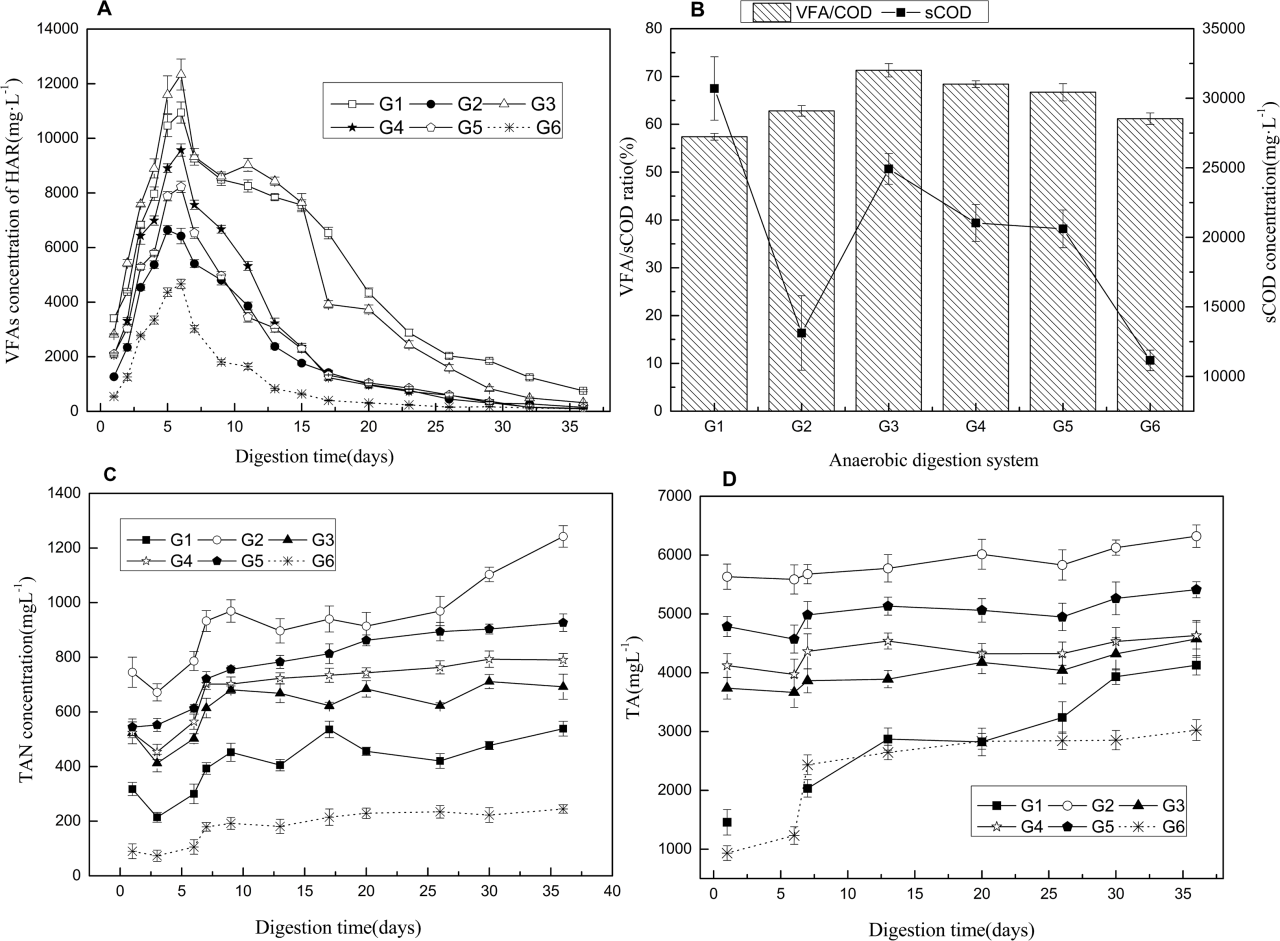
Total volatile fatty acids concentration (A), soluble COD and VFAs/sCOD ratio at sixth day (B), TAN concentration changes (C), total alkalinity (D).

In order to accurately depict the synergism in two-phase co-digestion, the additional methane yield over the weighted specific methane yield (WSMY) was introduced before [30]. It can be seen from Table 2 that the TMY of co-digestions were showed to be a positive differential from WSMY, and the TMY of G1-G5 were increased by 35.8, 17.3, 73.1,45.2 and 41.6%, respectively, compared with the corresponding WSMY. What's more, the differential of G3 containing 40% KW and 10% DD was higher than G1 which contained 50% KW. Therefore, the enhanced biogas production did not just attribute to the increased content of readily biodegradable substrates in the feedstock. It can be seen in Table 2, that the C/N ratio of mono-ORS digestion was 50.4, which was not in optimal range of 15–30 [8]. However, the C/N ratios of co-substrates in G1 to G5 were decreased to 37.5, 30.5, 36.1, 33.9 and 31.9, respectively. The decrease of C/N ratio could increase nitrogen nutrition in HAR for the hydrolytic bacteria, acidifying bacteria and methanogenic archaea [14]. Ammonia-nitrogen is not just a good nitrogen source for microbes, and it could affect the alkalinity. As can be seen from Fig 2C, the TAN concentration increased with the decreasing of C/N ratio, and then the buffering capacity of the AD system was improved, which eventually lead to the promotion of BY and MY [14, 25].

**Fig 3:**
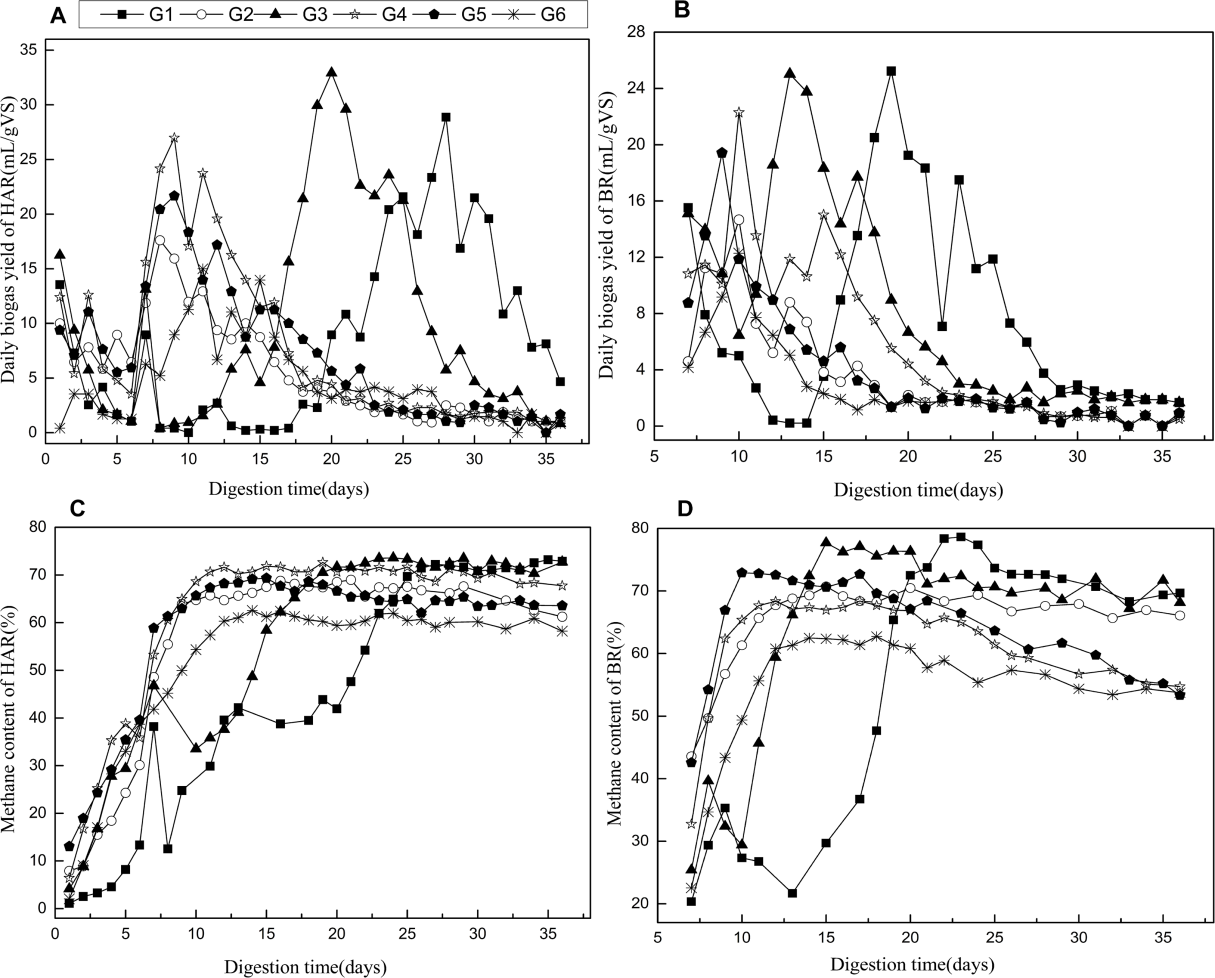
Daily biogas production of HAR(A), Daily biogas production of BR(B), Methane content of HAR(C), Methane content of BR(D) of different feedstock in two-phase mono- and co-digestion.

**Fig 4:**
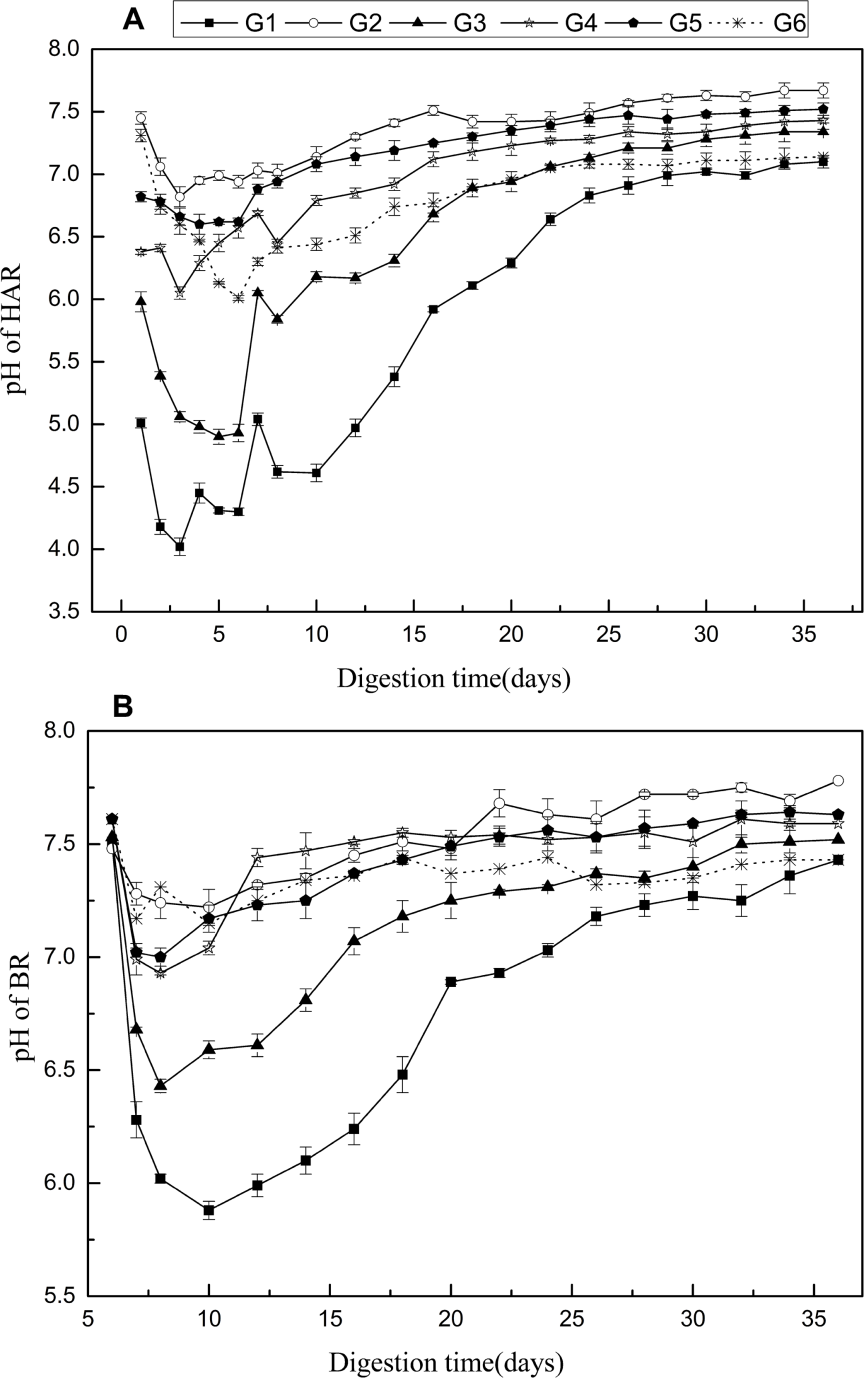
pH changes in HAR (A), pH changes in BR pH (B).

For different mixing ratio of feedings, the daily biogas yield in HAR, BR and the corresponding CH_4_ contents during the co-digestion are shown in Fig 3. It can be seen from Fig 3A, all the co-digestions (G1 to G5) were produced large amounts of biogas in the first day and dropped gradually until the 6th day, and similar changes appeared in G6 from the second day with lower biogas yields. This is mainly because the substrates were hydrolyzed and used by the anaerobic microorganisms and gradually inhibited by the low pH (see Fig 4A), and the ORS was hydrolyzed slower than co- substrates at start-up [31]. The HAR G2, G4 and G5, with 50, 25 and 40% DD in the feeding co-substrates reached the daily biogas production peak at around 10th day and earlier than G6. Thereafter, the daily biogas yield dropped gradually until the end of the trails. On the contrary, the co-digestions of G1 and G3 containing 50 and 40% KW both had a lag phase in HAR, which were 14 and 8 days, respectively, before reaching the peak of daily biogas yield. This could be due to more easily acidified organic matter caused lower pH (Fig 4) and inhibited the bio-gasification subsequently. As shown in Fig 3B, the BRs of G2, G4 and G5 showed no apparent inhibition to biogas production. On account of inoculum was acclimatized before fermentation, VFAs can be quickly consumed by methanogens in BRs, and thereafter reduced the VFAs inhibition in HARs. However, lag time (3 days) and significant inhibition were appeared in G1 and G3, respectively, both of which were self-recovered before the 15th day. This was due to low pH in HAR of G1 and G3 has significant impact on corresponding BR when hydrolysis-acidogenesis liquid pumped in [32]. The results reported by Ye et al. [17] showed the occurrence of inhibition when KW proportion was higher than 12% in the single-phase co-digestion of KW, pig manure and rice straw, some of which cannot recover even after pH adjustment. This indicated that two-phase AD had better buffering capacity in co-substrates digestion which contained easily acidified materials than the single phase digestion. The peak daily biogas yield in HAR and BR of G1 to G5 were all improved with the increase of KW proportion, but HAR of G3 was slightly higher than G1. This might be caused by the VFAs concentration in HAR of G1 was lower than G3, which contained three kinds of feedstock.

The methane content of biogas produced from HARs and BRs is shown in Fig 3C and 3D, respectively. The methane content in the different HARs and BRs reached steady state around the 9th day, and the methane percentage was 50–70% in the HAR and 55–78% in the BR. However, for the G1 and G3, the methane content in the HAR achieved steady state at the 20th and 15th day, respectively, and methane content of BR achieved steady state at the 13th and 23th day, respectively. The highest methane content of HAR and BR both obtained in G3 was 72.8 and 78.6%, respectively. All the methane content of BRs was higher than that in corresponding HARs, mainly because of the hydrolysis/acidogenesis process in HARs. The hydrogen content not measured in this study mainly because the hydrogen production will decrease with the pH gradually increase in HAR.

The ratio of methane yield from HAR and BR could illustrate the phase separation degree between hydrolytic-acidification phase and biogasification phase. Table 2 shows that the ratios decreased with the increase of KW proportion and with reduce of DD proportion in feedstock. This could be due to the growth and metabolism activities of the methanogens were inhibited by low pH and poor nutritional structure (C/N) in HARs, which contained higher proportion of KW [33]. In contrast, the increase of DD content could provide optimal nutritional supplement in HARs and help remove the inhibition and therefore more biogas obtained from HARs.

As a matter of fact, the methane obtained from HAR/BR ratio of individual ORS and DD digestion were prominently higher than other groups. A considerable amount of microorganism in animal manures [34] could improve the hydrolysis and gas generation process. ORS in HAR could be used as biodegradable carriers, which supported the growth of microorganisms [35], intercepted the methanogens in BR to form bacteria colloid, and thus increased the biogas yield in HAR. This may explain why more biogas was obtained from HAR compared to BR (Table 2). In addition, methanogens cannot be completely separated from acidogenic phase, and the inhibition of the methanogens in HARs was removed gradually along with the digestion [32].

### 3.4 Effect of different co-substrates on hydrolysis-acidogenesis liquid

#### 3.4.1 pH and VFAs concentration

Several reports suggested that the optimal pH for hydrolytic and acidogenic bacteria activity is between 5 and 6 [26, 36], and the proper pH for the methanogenesis is within the range of 6.8–7.2. For the pH in the HARs of G1-G5 decreased from the 1st to 6th day, and the values first dropped to 4.30–6.99, and then raised to 7.0–7.5 gradually. By contrast, the pH in BRs of G2, G4 and G5 were close to the neutral (from 6.93 to 7.78) during the entire period of digestions, and slightly decreased at the 7-9th day. However, pH in BR of G1 and G3 dropped to 5.88 and 6.43, respectively, and start to recover gradually from day 9–17 and day 10–20, respectively. Zhu et al. [37] investigated the biogas yield from municipal solid wastes, the result shown that the pH of HAR (7.3–7.8) was very close to that in BR (7.9–8.1), but the pH showed different trend between HAR and BR in this study. It is due to less inoculation sludge was added in HAR in this study, and VFAs in HAR was consumed slower by methanogenic bacteria compared with Zhu’s study. The varying degrees of pH recovery were observed in the different co-digestions, which contained 25–50% DD and recovered rapidly to obtain over 80% biogas before the 20th day. The alkaline matters in DD and the NH_4_^+^-N production during the digestion process might have neutralized the pH, and thus enhanced the buffer capacity of two-phase anaerobic system.

On the other hand, the presence of large proportion of DD in the co-substrates could cause the decrease of other bio-degradable materials, and led to the decrease of total biogas and methane yields. It can be seen from Fig 4A and Table 2 that HAR of G3 had suitable pH for hydrolytic and acidogenic bacteria, and the corresponding BR have relatively short recovery time compared to that in G1. However, previous studies showed that the anaerobic co-digestions caused the failure of digestion or chemicals were needed to adjust pH when the proportions of KW were high [17, 36]. This proved that the BR of the two-phase co-digestion system presented the high buffer capacity to avoid acidification during the anaerobic digestion even if an accumulation of VFA occurred.

For the group G1-G6, the VFAs concentrations existing in HARs increased in the first 6 days and decreased abruptly after HARs connected to BRs, and then gradually decreased until the end of the operation (Fig 2A). It is generally known that the growth and metabolism rate of hydrolytic bacteria and acidifying bacteria are faster than methanogenic bacteria, thus causing VFAs accumulate at the beginning. When HARs and BRs were connected, the methanogenic bacteria in BRs started to consume VFAs, and some of which were transferred into the HARs. The VFAs production and consumption kept relatively stable to the end.

The analyses of total volatile fatty acids (TVFAs) concentration of HARs (Fig 2) showed that higher TVFAs concentration existed in the HARs of G1-G5 than in the pure ORS reactors, and there is an increasing trend with the increase of the KW content. This suggested that the addition of KW and DD could promote the TVFAs production in HARs. However, the G1 which contained 50% KW obtained lower (*p<*0.05) TVFAs concentration than G3 (KW: 40%, DD: 10%). The TVFAs concentration was affected by the KW content mainly due to rapid hydrolysis and acidification of the KW in the first 5–6 days. In addition, the DD added as co-substrate caused less inhibition of hydrolysis and acidification. A similar result was obtained during the single-phase co-digestion of rice straw, kitchen waste and pig manure [17].

As shown in Fig 4A and Fig 2A, the decrease of pH was mainly caused by the accumulation of VFAs. The total VFAs concentrations in all HARs reached the peak values ranging from 6000 to 13000 mg/L during the initial 5–6 days, and were significantly affected by the proportion of KW in the co-substrates. This could be due to the KW, containing high contents of organic matters, which can be easily converted to VFAs and cause low pH. Similar findings were reported by Ye et al. [17, 36]who noticed that maximal VFAs concentrations occurred in the single-phase co-digestion with the highest proportion of KW. However, the VFAs concentrations and pH of G1 were both lower (*p*<0.05) than those in G3 (Fig 2A). This indicated that the addition of DD could improve the buffering capability and acidification efficiency in HAR once again.

#### 3.4.2 VFAs/sCOD and VFAs distribution

In the acidogenic phase of two-phase anaerobic co-digestion, the ratio of VFAs/sCOD can indirectly characterize the acidification degree of the materials. As can be seen from the Fig 2B, VFAs/sCOD ratio were somewhat increased in the co-digestions compared with the ORS mono-digestion except in G1. The maximal ratio of 71.3% was obtained in G3, which was higher than that in G2, G4, G5 and G6 at p<0.05 level, and higher than that in G1 at p<0.01 level. The VFAs/sCOD ratio obtained in G2 was not significantly (*p*>0.05) improved than that obtained in G6, which suggested that the addition of both individual KW or DD could not improve the acidification efficiency. These can be attributed to more suitable pH and nutrition (Fig 4A) for acidogenic bacterial activity in the HAR with three kinds of substrates. Previous studies reported the lower VFAs/sCOD ratio in the HAR of (co-)digestion mainly because of unsuitable pH and excessive organic load [33, 38]. As can be seen from Fig 2B, sCOD existing in HARs of G1-G6 shows a rising trend with the increase of KW proportion. The corresponding TVFAs concentrations obtained at the 6th day ranged from 5359.2 to 17768.6 mg COD/L (Table 3), which shared the similar trends as when sCOD was affected by KW contents. As reported before, the salinity and fat in KW can inhibit the microbial activity of acidifying bacteria, which might be the reason for lower VFAs/sCOD ratio and relative higher sCOD obtained in G1 than G3 [39].

**Table 3:** Individual VFA contents in the mono- and co-digestion of HARs.

Group	TVFAs mgCOD/L	Distribution of VFAs					
		acetic acid %	propionic acid %	isobutyric acid %	butyric acid %	valeric acid %	isovaleric acid %
G1	17620.9	24.6	31.5	0.3	38.3	4.8	0.5
G2	8241.3	64.3	18.4	3.2	11.9	1.9	0.3
G3	17768.6	30.3	29.8	0.6	37.0	2.2	0.2
G4	14387.0	46.1	20.7	2.6	27.3	3.2	0.4
G5	13749.7	46.7	13.9	2.0	33.1	4.2	0.6
G6	6823.6	45.2	12.5	3.0	29.7	8.6	1.1
G7	12328.8	27.6	50.2	3.0	14.5	3.4	0.9
G8	5359.2	59.4	20.0	5.0	12.0	2.1	1.6

On account of the increasing trend of TVFAs was became gentle after the 6th day based on pre-experimental, and the 6th day was chose to discussed in this study. The TVFAs obtained at the 6th day were calculated by sum of acetic, propionate, iso-butyric, butyric, valeric and iso-valeric acid, and the distribution of VFAs in different co-digestions are shown in Table 3. Acetic acid, propionic acid and butyric acid composed 87.4–97.1% of VFAs, which played an important role in producing methane during anaerobic co-digestion. According to Zhu et al. [37], acetic acid and butyric acid were the most important VFAs in the HARs, which were normally produced by hydrolysis/acidogenesis fermentation and could be easily converted to methane by anaerobic microorganisms. This is mainly because acetate acid could be directly converted to CH_4_ and CO_2_, but butyric acid and propionic acid needs intermediate conversion to form acetate first. The standard Gibbs free energy change of these three processes are -31.0, +48.1 and +76.1 kJ/mol, respectively. Therefore, compared to butyric acid, propionic acid can be converted to acetic acid when the hydrogen partial pressure and acetic acid concentration were at lower levels [17].

As shown in Table 3, the acetic acid content in VFAs of the HARs (G1-G5) reached 24.6, 68.3, 42.3, 46.1 and 46.7%, respectively, and propionic acid content arrived at 31.5, 18.4, 22.8, 20.7 and 13.9%, respectively. The content of acetic acid in VFAs of different HARs showed increasing trend when the DD content increased and KW content decreased, and this trend was gentle between G4 and G5. While the content of propionic acid showed a contrary tendency. It is because of the high C/N ratio and easily degradable contents in the substrates (see Table 1), which caused low pH (see Fig 4). The pathway of acetic acid convert into methane was inhibited at low pH, and then the acid accumulation was appeared [40]. On account of feedback effects the conversion of propionic acid was inhibited. Similar results have been reported that propionic acid and lactic acid were the main products for the KW fermentation [41].

The propionic acid concentration in G3, G4 and G5 reached 3497.4, 1967.0 and 1262.4 mg/L, respectively, showing no obvious inhibition to methanogenesis of BRs. However, BR of G1, in which the propionic acid concentration achieved 3666.2 mg/L, showed unstable performance (Fig 3B), and did not showed the complete digestion failure. Pullammanappallil et al. [42]reported that the single-phase digestion would be inhibited seriously or cause the failure of system function when the propionic acid concentration was higher than 3000 mg/L. Based on these results, it could be concluded that the two-phase co-digestion of ORS, KW and DD present better capability to remove inhibition. Nevertheless, the potential risk of propionic acid inhibition to methanogenesis could occur in the two-phase anaerobic co-digestion when KW proportion was higher.

#### 3.4.3 Ammonia nitrogen and alkalinity

The variations of total alkalinity (TA) and total ammonia-nitrogen (TAN) during the digestion period are shown in Fig 2C and 2D. It is generally known that ammonia-nitrogen (NH_4_^+^-N) can combine with carbon dioxide in water to form ammonium bicarbonate, which is attributed to the alkalinity and help maintain the pH value. As can be seen in Fig 2C, TAN concentrations of all the co-digestions decreased slightly during the early phases in HARs. Subsequently, they began to rise and remained stable between 538.5 and 1242.3 mg/L. Compared with mono-digestion with individual ORS, the TAN concentration in the fermentation liquor of co-digestion was obviously higher (*p*<0.05). Ammonia-nitrogen was produced from the hydrolysis process of organic nitrogen compounds at the beginning of digestion, and the propagation of microorganisms consumed certain nitrogen. The TAN may increase again when the microorganism density was stable and the organic nitrogen in the feedstock kept hydrolyzed slowly.

The function of alkalinity in fermentation liquor, which primarily is composed of carbonate, bicarbonate and hydroxide, is to bind hydrogen ions. For the two-phase anaerobic digestion, methanogenic effluent provided the bicarbonate alkalinity for pH buffering in the HAR. These alkaline materials, which were generated during the fermentation period, have capacity to buffer the acidic materials. As shown in Fig 2D, TA of co-digestions fluctuated in the range from 1500 to 6300 mg CaCO_3_/L, all the TA of co-digestions were instable in the early phase due to the VFAs accumulation, and gradually become steady with the consumption of VFAs. The maximal TA obtained in G2 reached 6321 mg CaCO_3_/L. With the dissolution of organic nitrogen from DD and KW, the TAN concentrations increased to neutralize the VFAs in acidification and increase the alkalinity. On the other hand, methanogenic effluent provided the bicarbonate alkalinity and relieves the inhibition of VFAs in HARs.

Both concentrations of TAN and TA increased with the increase of DD proportion in the feedstock under all different co-digestion conditions, and the corresponding pH increased at the same time (Fig 4). Higher DD content in the substrates provided higher concentrations of ammonia and may be the main source for buffering capacity improvement. A previous study on the two-phase digestion of kitchen waste suggested that enough alkali was required for pH buffering, and NaOH was therefore added to control the pH [27].

According to the results in this study, the addition of KW and DD in two-phase co-digestion process induced high ammonia concentration and improved the buffering capacity of the system. However, it is hard to reduce inhibition from ammonia in the two-phase co-digestion when the feedstock contained high DD contents [10, 13]. What is more, excessive DD content in feedstock may cause poor phase separation and low methane yield (Table 2). Therefore, considering the TVFAs concentration and total methane yield in each kind of two-phase co-digestion, the optimal mixing proportion of ORS, KW and DD are 50:40:10 when maximal TVFAs contents, the total biogas yield and the total methane yield were obtained. Meanwhile, the obvious inhibition to methanogenesis by VFAs in BR did not occur.

### 3.5 Biodegradation of ORS

**Fig 5:**
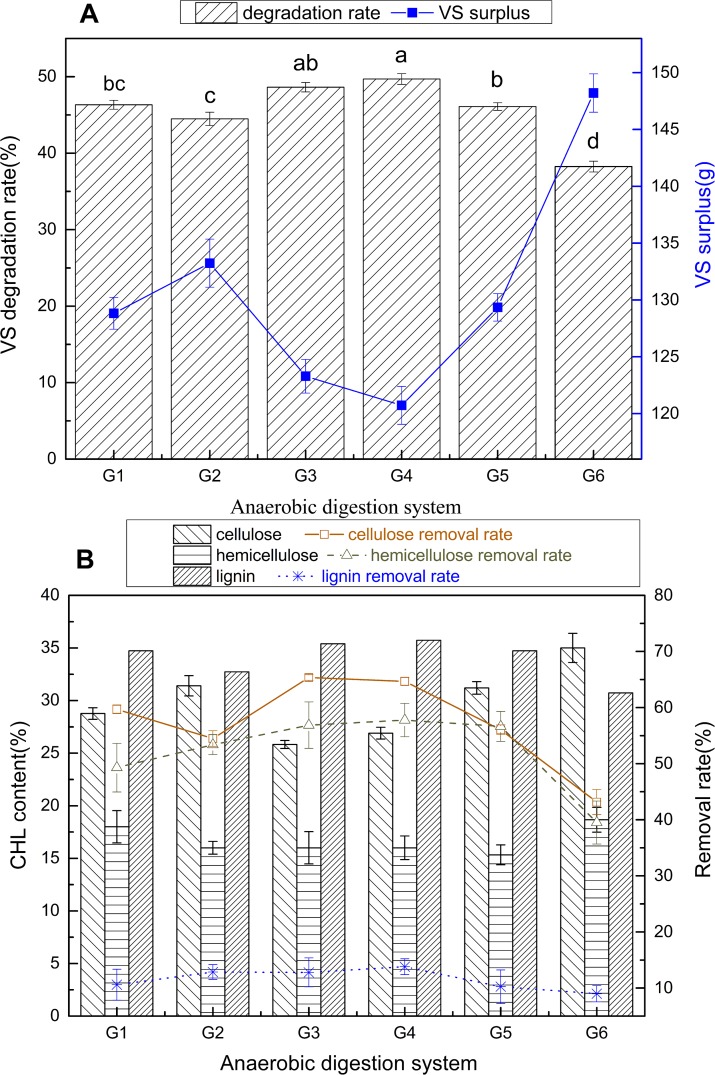
**Biodegradation of ORS after two phase anaerobic digestion process: A. VS degradation rate and surplus, B. cellulose, hemicellulose and lignin content in fermented oilseed rape straw.** CHL: cellulose, hemicellulose and lignin. The mean values were calculated from three repeats, and standard deviations were represented by vertical bars. Different letters mean significant difference with a probability p < 0.05.

The organic matters in ORS mainly consisted of cellulose, hemicellulose, lignin, proteins, pectin and fat. In this study, the cellulose, hemicellulose and lignin (CHL) composed of 86.3% the dry matter in ORS (Table 1). As shown in Fig 5A, the main organic content (VS content) of the ORS was degraded in the two-phase anaerobic co-digestion. It is known that those solid organic substrates containing abundant lignocelluloses always have low biogas yield due to the low hydrolysis rate in HARs. Therefore, hydrolytic-acidification process could be considered as the rate-limiting step of ORS biodegradation.

Fig 5A shows that the VS degradation rate of ORS in the two-phase mono-digestion (G6) is increased from 16.3 to 27.1% compared with the two-phase co-digestions. According to the statistical analysis, the VS degradation rate obtained in G3 and G4 of 48.6±0.6 and 49.7±0.7%, respectively, was no significantly different (*p>*0.05), but significantly different from that in G1, G2 and G4 (*p*<0.05). All of the co-digestions were very significantly improved (*p*<0.01) on VS degradation rate compared to individual ORS in two-phase AD. No matter single co-substrate (G1, G2) or two co-substrates (G3, G4 and G5) were added in two-phase AD, the ORS has better biodegradability than with only individual ORS (G6). These results showed the similar trends with previous research on the co-digestion of chicken manure with cereal residues [43], and kitchen waste with straw [17]. These should be attributed to several reasons: firstly, the hydrolytic and acidification efficiency had a high level when the pH was at 6.0 [27], which improves the conversion of organic solid in ORS into dissolved organic matter; secondly, kitchen waste addition could improve the cellulase activity compared with the individual ORS fermentation [7]; thirdly, the C/N ratio of individual ORS was not suitable for the hydrolyzing and acidifying bacteria, and DD addition could balance the C/N ratio and provide nitrogen source for the microorganisms growth in AD process [44]. Additionally, further exploration is needed to find the relationship between dynamics of microbial community and variation of the ORS: KW: DD ratio.

As can be seen in Fig 5B, the contents of cellulose and hemicellulose in the ORS in G1 to G5 were lower than those in G6 after 36 days’ fermentation, and all the lignin contents were higher than the raw ORS. The removal rate of cellulose (43.1–65.4%) and hemicellulose (39.5–57.7%) were higher than that of lignin, leading to the relative increase of lignin contents in fermented ORS. Cellulose removal rate of the ORS in two co-substrates addition system (G3, G4, G5) were higher than single co-substrate addition system (G1, G2), showing the similar trend as the VS degradation rate. It can be concluded that cellulose removal rate increased with the increase of KW content, and the two co-substrates addition system (G3, G4, G5) obtained higher cellulose removal rate than the G1 and G2. However, the removal rates of the hemicellulose and lignin were not significantly affected by different co-digestions. The loss of lignin might due to solubilization of low molecular weight lignin and lignin particles suspend in liquid [45]. These results indicated that cellulose and hemicellulose in ORS are the main components converted to VFAs and methane, and the addition of KW and DD as co-substrates could significantly improve (*p*<0.05) the cellulose consumption rate compared to that in two-phase mono-digestion.

## 4. Conclusions

Two-phase co-digestion of ORS with KW and DD as co-substrates was a promising approach to improve the system stability, biogas and methane yields. Poor methane production rate and the system stability were found in mono-digestion of ORS, KW and DD, due to high lignocelluloses content, low buffering capacity and low C/N ratio, respectively. The co-digestion of ORS, KW and DD could increase methane yield which is attributed to the improvement of buffering capacity by DD and the increased hydrolysis-acidification by proper pH. Besides, the addition of KW and DD as co-substrates with ORS in two-phase AD significantly improved the biodegradability of ORS.

## Abbreviations

ORS: Oilseed rape straw
KW: Kitchen waste
DD: Duck droppings
AD: Anaerobic digestion
TMY: Total methane yield
HAR: Hydrolytic-acidification reactor
BR: Biogasification reactor
TVFAs: Total volatile fatty acids
VFAs: Volatile fatty acids
BY: biogas yield
MY: methane yield
TMP: Theoretical methane potential
WAMC: Weighted average methane content
WSMY: Weighted specific methane yield
CHL: Cellulose, hemicellulose and lignin
MC: Moisture content
TS: Total solids
VS: Volatile solids
sCOD: Soluble chemical oxygen demand
TAN: Total ammonia nitrogen
TAN: total ammonia-nitrogen
TA: Total alkalinity
SD: standard deviation
ANOVA: One-way analysis of variance
